# An objective analysis of quality and readability of online information on COVID-19

**DOI:** 10.1007/s12553-021-00574-2

**Published:** 2021-06-24

**Authors:** N. E. Wrigley Kelly, K. E. Murray, C. McCarthy, D. B. O’Shea

**Affiliations:** grid.412751.4 0000 0001 0315 8143 St. Vincent’s University Hospital, University College Dublin, Dublin, Ireland

**Keywords:** COVID-19, Coronavirus, Information, Quality, Readability, Internet

## Abstract

High quality, readable health information is vital to mitigate the impact of the COVID-19 pandemic. The aim of this study was to assess the quality and readability of online COVID-19 information using 6 validated tools. This is a cross-sectional study. “COVID-19” was searched across the three most popular English language search engines. Quality was evaluated using the DISCERN score, Journal of the American Medical Association benchmark criteria and Health On the Net Foundation Code of Conduct. Readability was assessed using the Flesch Reading Ease Score, Flesch-Kincaid Grade Level and Gunning-Fog Index. 41 websites were suitable for analysis. 9.8% fulfilled all JAMA criteria. Only one website was HONCode certified. Mean DISCERN score was 47.8/80 (“fair”). This was highest in websites published by a professional society/medical journal/healthcare provider. Readability varied from an 8th to 12th grade level. The overall quality of online COVID-19 information was “fair”. Much of this information was above the recommended 5th to 6th grade level, impeding access for many.

## Introduction

Coronavirus disease 2019 (COVID-19) was first described in Wuhan, China in December 2019. As of June 3rd 2021 it has caused more than 3.5 million deaths, with 171 million confirmed cases [1]. In addition this pandemic has severely impacted the global economy, the World Bank predicting that per capita income will contract in the largest fraction of countries internationally since 1870 [2]. Measures to ameliorate the morbidity, mortality and economic burden of this disease – including social distancing approaches, hand washing, mask wearing and vaccination—are contingent upon the availability of high quality, readable health information which is readily accessible.

Historically healthcare professionals have represented the primary source of health care information for the public. However, recent years have seen major changes in terms of the availability of health information through other sources. There has been an increase in internet usage worldwide, as well as an increase in the depth and breadth of online content [3, 4]. The internet is now among the most common sources of health care information for patients [5–7]. While the availability and usage of online health information has expanded, the quality and readability of that online health information varies considerably [8]. A number of validated tools assess both the quality (*Journal of the American Medical Association* [JAMA] benchmark criteria, DISCERN criteria, HONcode certification) and readability (Flesch Reading Ease Score [FRES], Flesch-Kincaid Grade Level [FKGL], and Gunning-Fog Index [GFI]) of online healthcare information.

Guidelines specify health information intended for consumption by the general public should be at a 5th to 6th grade reading level (ages 10 to 12) [9]. Previous studies of other medical conditions have found most online health care information to be above this level, impeding access for many people [10–14]. The only published study assessing the readability of online COVID-19 information to date found the information to be at an 11 to 14-year-old reading level [15]. However, there were limitations to this study; only one internet search engine (Google) was assessed and importantly the study did not assess information quality. The objective of our study was to assess both the quality and readability of current online health information regarding COVID-19, using six previously validated tools and to compare this information with published guidelines.

## Methods

### Internet search strategy

We identified the most featured English language search terms pertaining to this disease: “*COVID-19”*, “*2019-nCoV”*, “*novel coronavirus”,* “*COVID”, “Coronavirus”, “Coronavirus disease” and “SARS-CoV-2”*. Those terms were searched across Google, Bing, and Yahoo! These search engines account for over 97% of United Kingdom (UK) searches [16]. *“COVID-19”*, the search term leading to the largest number of web addresses, also known as Uniform Resource Locators (URLs), was selected for analysis. Given the previous evidence showing that patients rarely search beyond 25 pages [17], the most-viewed 25 URLs on each search engine were included for analysis [18]. While the authors of this study are based in Irish centres, the UK was set as the jurisdiction for search engines, thereby allowing for broader generalizability of results.

Inclusion criteria were the first 25 URLs from each search engine. Duplicate websites, websites providing financial information as opposed to healthcare information and non-readable sites (non-text and pay wall protected pages) were excluded. In the event of a single item being spread across sequential pages on the same website (pagination), the sequential pages were also assessed. All websites were reviewed from July 13th to 17th 2020.

The website producer (group responsible for hosting or publication of the website) was categorized as governmental organisation, non-governmental organisation, for profit organisation and professional society/medical journal/healthcare provider. Explicit naming of authorship was required for websites to be seen to be compliant with that aspect of the JAMA guidelines. Where websites detailed dates for both content creation and time of last content, the most recent date was used for evaluating website currency.

### Assessment of quality

Website quality was assessed through three well established, validated tools: JAMA benchmark criteria, DISCERN criteria and Health On the Net (HON) Foundation Code of Conduct (HONcode) certification [10, 11, 18].

The JAMA benchmark criteria consist of 1) identification of authorship, 2) identification of sources, 3) specifying the date of creation/update, and 4) disclosures (of ownership, advertising policy, sponsorship, and conflicts of interests) [19]. The presence or absence of each criterion was recorded. The content producer parameter was taken from the webpage itself or the Contact Us/About Us section/link.

DISCERN is an instrument that assesses website quality and reliability by grading 16 items (concerning reliability, description of treatment choices, and overall rating) from 1 (inferior) to 5 (superior). Websites are scored from 16 to 80, with a higher score indicating better-quality information [20].

HON is a non-profit organisation linked to the Economic and Social Council of the United Nations with the stated aim of enhancing the dissemination of quality health information globally [21]. Evaluation involves assessing for disclosure of authors’ qualifications, attribution/citation of sources, data protection, justifiability, transparency, and disclosure of sources of funding and advertising. More than 8,000 sites have been certified [22]. In this study certification was checked for each website by cross referencing with an HONcode database.

### Assessment of readability

The readability of each website was evaluated via three scoring systems: Flesch Reading Ease Score (FRES), Flesch-Kincaid Grade Level (FKGL), and Gunning-Fog Index (GFI) of online healthcare information. All three scores are calculated via an online analysis tool [23], therefore allowing for objectivity. Their results are interpreted according to the years of education typically required for that level of literacy or readability [24].

Rudolph Flesch developed the Flesch Reading Ease Score (FRES) in 1948. It involves calculation of readability using the formula 206.835 − 1.015*(total words/total sentences) − 84.6*(total syllables/total words). For example, for a website that contains 90 sentences, 383 words and 597 syllables, the calculation would be as follows: 206.835 − 1.015*(383/90) − 84.6*(597/383) = 70.6. Higher scores indicate easier readability [25]. The FRES reading score is widely used, for example being the standard readability test employed by the US Department of Defense [26].

The FKGL score was originally developed for the United States Navy in 1975 as a means of evaluating the readability of military manuals. It assesses readability with the following formula: 0.39*(total words/total sentences) + 11.8*(total syllables/total words) − 15.59 [27]. As detailed in the formulae above, the FRES and FKGL use the same core measures (word and sentence length), however they differ in terms of the weighting of the individual factors, FKGL emphasising sentence length over word length.

GFI assesses readability with the formula 0.4 x ([words/sentences] + 100 x [complex words/words]).

The FKGL and GFI formulae both produce scores as US educational system grade levels, thereby being more readily interpretable than scores produced by FRES. By way of basic guide, US grade 1 approximates to ages 6–7, while grade 12 corresponds to ages 17–18 [28].

These readability scores were chosen as they are validated systems which have been shown to be consistent in terms of their results [29, 30], while also being easy for content makers to use. FRES and FKGL are the most widely used readability scoring systems, therefore allowing for broad comparability and generalizability of results [30, 31]. The GFI was selected as it provides a further level of nuance to the analysis of readability. It factors in word complexity and unfamiliarity through the use of a list of common words that despite having a relatively large syllable count are not considered to be complex. Another readability score of merit although not included in this analysis is the Simple Measure of Gobbledygook (SMOG). The SMOG is also easy for content makers to use, however it focuses solely on polysyllabic word content [29]. Therefore, this score lacks a degree of complexity seen in the other three, analysing text on lexical but not syntactic grounds. Lastly, it is worth highlighting that no one scoring system has been widely recognized as a gold standard for the assessment of readability. Therefore the use of multiple, validated scoring systems in this fashion allows for a balanced and holistic evaluation.

### Statistical methods

Data analyses were performed using Prism 7 (GraphPad software, San Diego, CA, USA.). Normally distributed continuous data are presented as the mean and standard deviation [32]; non-normally distributed data are presented as the median and interquartile range [33]. Website readability and quality scores for each website were analysed with one-way analysis of variance (ANOVA) and Kruskal–Wallis test, as appropriate. Significance was set at *p* < 0.05.

## Results

### Internet search strategy

On July 13th 2020 the search terms *“COVID-19”*, *“2019-nCoV”*, *“novel coronavirus”*, *“COVID”*, *“coronavirus”*, *“coronavirus disease”* and *“SARS-CoV-2”* were searched across the three most commonly used English language search engines. The search term *“COVID-19”* provided the most results, with a combined total of 13,827,000,000. The three next highest scoring search terms were *“COVID”* (13,778,000,000), *“novel coronavirus”* (6,336,000,000) and *“2019-nCoV”* (5,382,220,000). The search term *“COVID-19”* was therefore selected for analysis.

**Fig. 1:**
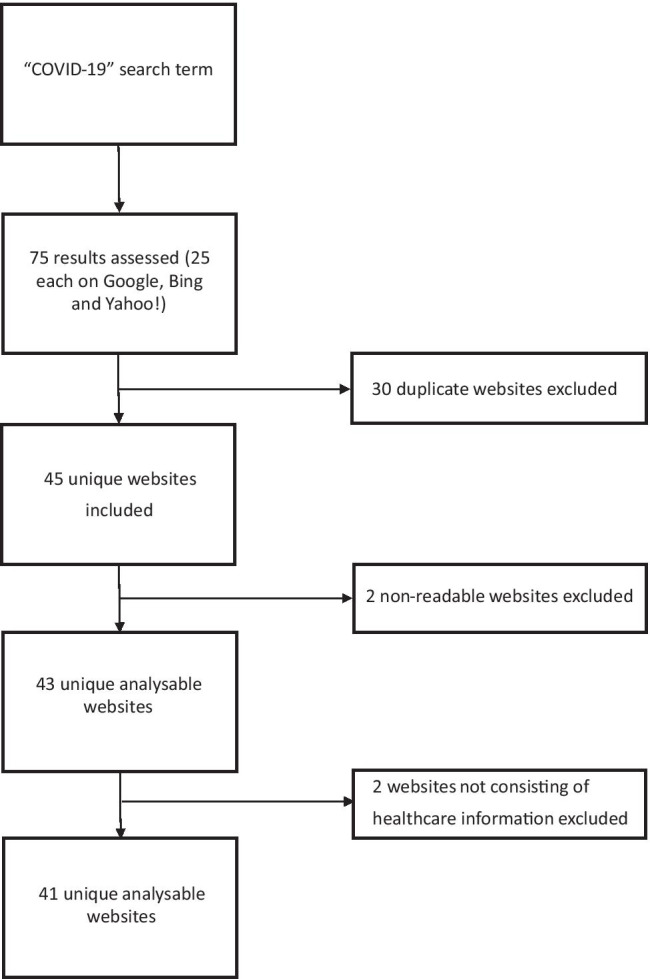
Internet search strategy for COVID-19 search term

The 25 top-ranking websites from each search engine were initially included. Of these 75 websites, 34 were excluded. These were 30 duplicate websites, 2 websites providing financial information as opposed to healthcare information, 2 non-readable websites (non-text pages [n = 1] and pay wall protected websites [n = 1]). Therefore, 41 websites were included for analysis. The internet search strategy is summarized in Fig. 1.

Of the 41 websites accessed 17 were governmental organisations. Given the UK search settings, unsurprisingly there was a predominance of UK sites among these (n = 8). Otherwise there was a relatively even distribution, albeit with mostly Anglophone countries featuring, including two United States governmental sites, two European Union and one each from Canada, Australia, New Zealand, South Africa and Nigeria. There were 10 non-governmental organization sites, including the World Health Organization, Wikipedia and Afghanaid. All except one of the 7 for profit corporation websites were news outlets, including the Daily Mail, CNN and the South China Morning Post. The 7 sites in the professional society/medical journal/healthcare provider category also accounted for a relatively broad geographical distribution, including the Lancet, the European Respiratory Society and Johns Hopkins Medical.

### Quality

Six of 41 (9.8%) websites fulfilled all four *JAMA benchmark criteria*: four from the professional society/medical journal/healthcare provider category and two non-governmental organisations.

Overall, the name of the website author was reported in 14/41 websites (34.1%). 22/41 (53.7%) were compliant with the attribution criterion (listing of references and sources for content), website ownership, sponsorship, advertising and commercial funding arrangements were disclosed in 20/41 websites (48.8%), while the website currency criterion (listing of dates at which content was posted and updated) was adhered to in 39/41 websites (95.1%).

JAMA score varied significantly according to publishing organisation type (H(3) = 11.27; p = 0.0103). Websites produced by governmental organisations had a significantly lower score (median = 2) compared with those produced by professional societies/medical journals/healthcare providers (median = 4) (p = 0.0077). Pairwise comparisons did not otherwise identify significant differences (see Table 1).

**Table 1:** Quality of COVID-19 online information by website producer type

Producer	Mean DISCERN score	Fulfill JAMA^1^ benchmark Criteria, n (%)			
		Authorship	Attribution	Currency	Disclosure
All (n = 41)	47.8	14(34.1)	22(53.7)	39(95.1)	20(48.8)
Governmental organisation (n = 17)	47.3	0(0)	6(35.3)	17(100)	7(41.2)
Non-governmental organisation (n = 10)	45.2	2(20)	6(60)	8(80)	9(90)
For profit organisation (n = 7)	41.9	6(85.7)	4(57.1)	7(100)	0(0)
Professional society/medical journal/healthcare provider (n = 7)	58.4	6(85.7)	6(85.7)	7(100)	4(57.1)

^1^JAMA: Journal of the American Medical Association

**Fig. 2:**
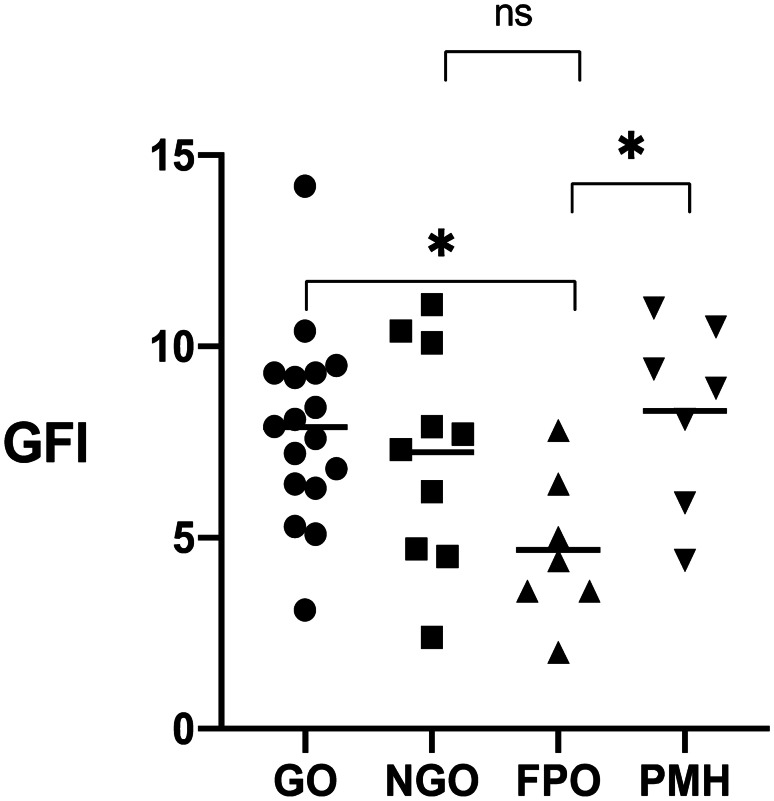
Mean GFI (Gunning-Fog Index) (range 0–20) according to website producer organisation type. Individual values are shown with mean represented by bar. ns = Not significant, * = p < 0.05. GO = Governmental organisation, NGO = Non-governmental organisation, FPO = for profit organisation, PMH = professional society/medical journal/healthcare provider

Overall mean DISCERN score across all websites was 47.8 or “fair” [34]. The website with the highest DISCERN score was the Wikipedia ‘COVID-19 pandemic’ page, with a score of 68 (“excellent”) [35]. This site fulfilled 3 of 4 JAMA benchmark criteria but was not HONcode certified. Of note, it also had FRES, FKGL and GFI scores corresponding to 8th/9th, 6th and 7th grade reading level, respectively (see Fig. 2).

**Fig. 3:**
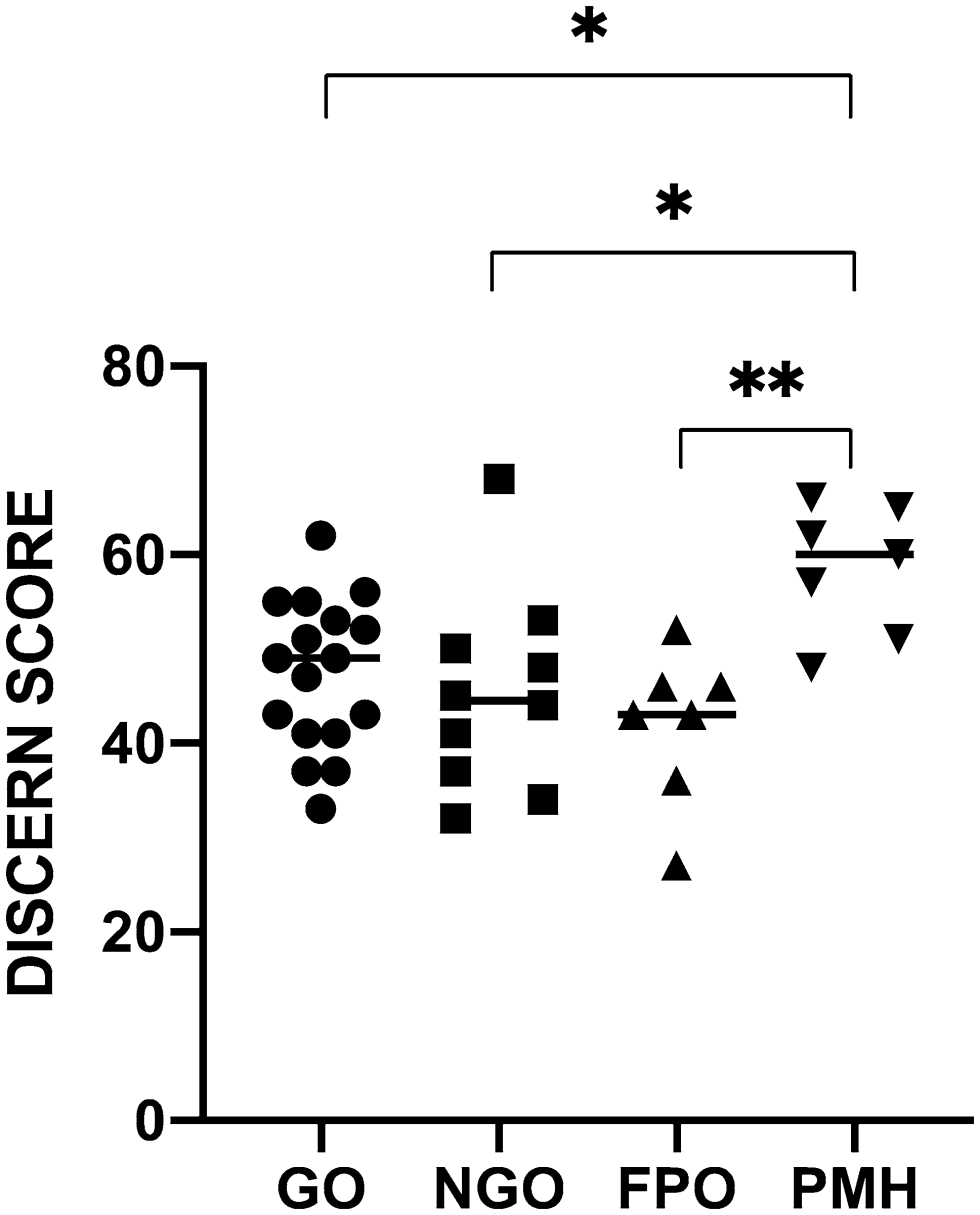
DISCERN score (range 16–80) by type of website producer. Individual values are shown with mean represented by bar. * = p < 0.05, ** = p < 0.001 GO = Governmental organisation, NGO = Non-governmental organisation, FPO = for profit organisation, PMH = professional society/medical journal/healthcare provider

Significant differences in mean DISCERN score were observed between publishing organisation types (ANOVA r^2^ = 0.2919; p = 0.0048). Websites of a professional society/medical journal/healthcare provider origin were seen to have a significantly higher DISCERN score (mean 58.4; SD ± 6.85) than those produced by the other 3 categories of publisher: governmental organisation (mean 47.3; SD ± 8; p = 0.0299), non-governmental organisation (mean 45.2; SD ± 10.5; p = 0.0164) and for profit organisation (mean 41.9; SD ± 8.1; p = 0.0045) (see Fig. 3).

Only one of the 41 websites (2.4%) was HONcode certified. This webpage was *NHS: Conditions—Coronavirus (COVID-19)*.[36]

### Readability

The mean FRES of all websites (n = 41) was 53.9 (SD ± 14.3), indicative of a 10th to 12th grade reading level, mean FKGL was 7.6 (SD ± 2.1) equating to an 8th grade reading level, while mean GFI was 7.3 (SD ± 2.7) also indicating an 8th grade reading level.

Mean GFI scores differed by the type of website (r^2^ = 0.2108; p = 0.031). The mean GFI of websites produced by for profit organisations (n = 7) was 4.69, equating to a 5th grade reading level. This was significantly lower than that seen for government organisations (p = 0.0324) and websites produced by professional societies/medical journals/healthcare providers (p = 0.0450) (Fig. 2). There were no significant differences in FRES reading level (ANOVA r^2^ = 0.009894; p = 0.9458) and FKGL (r^2^ = 0.007388; p = 0.9641) scores by organisation type (see Table 2).

**Table 2:** Readability of COVID-19 online information by website producer type

Producer	Mean readability score		
	FRES^1^	FKGL^2^	GFI^3^
All (n = 41)	53.9	7.6	7.3
Governmental organisation (n = 17)	52.7	7.5	7.9
Non-governmental organisation (n = 10)	56.2	7.6	7.2
For profit organisation (n = 7)	54.1	7.4	4.7
Professional society/medical journal/Healthcare provider (n = 7)	53.3	8	8.3

^1^*FRES *Flesch Reading Ease Score

^2^*FKGL* Flesch-Kincaid Grade Level

^3^*GFI* Gunning-Fog Index

## Discussion

In their 2011 report the Special Rapporteur of the United Nations Human Rights Council underscored “the unique and transformative nature of the internet not only to enable individuals to exercise their right to freedom of opinion and expression, but also a range of other human rights, and to promote the progress of society as a whole”. In doing so they specified the importance of supporting “initiatives to ensure that online information can be accessed in a meaningful way by all sectors of the population, including persons with disabilities and persons belonging to linguistic minorities” [37]. Given the health and economic implications of the global COVID-19 pandemic, counter measures to mitigate its effects are of particular importance. This approach is contingent on the availability of accessible, high quality public health information.

In 2017 the World Health Organization (WHO) published the guideline ‘Communicating risk in public health emergencies’ [38]. This work built on the aforementioned 2011 UN report by highlighting the implications that technological advances have on health information during public health emergencies. In particular, the guideline stresses the importance of “accurate information provided early, often, and in languages and channels that people understand trust and use”. The issue of trust has heightened importance in an age of "fake news" (information that mimics news media content in form but not in organisational process or intent) and other forms of information disorder, including misinformation (false or misleading information) and disinformation (false information that is purposely spread to deceive people) [39]. The features inherent in poor quality online health information—such as failure to identify authors/sources and omitting to disclose conflicts of interest—are precisely the circumstances in which these forms of false information prosper. Undermining trust and damaging the health of a community. We would advocate that governments and other parties who produce online health information have a responsibility not only to adhere to these standards of quality but to demand them of others in an organised fashion.

Overall this analysis showed that COVID-19 online information readability levels—as assessed by all three scoring systems—were greater than the recommended 5th to 6th grade level [9]. Therefore restricting access for a substantial proportion of the population. The overall quality of online COVID-19 information—as per the DISCERN score—is “fair”. Websites produced by professional societies/medical journals/healthcare providers generally scored significantly higher than all three other publishing organisation types. Websites produced by professional societies/medical journals/healthcare providers also met significantly more of the JAMA benchmark criteria than those produced by governmental organisations.

This study had limitations. While the readability evaluation tools FRES, FKGL and GFI are objective and reproducible for text websites, they do not analyse audio, image or video based information. This limitation has been identified in other similar studies [40]. Conversely, while website health information quality assessment tools are well defined, systematic and evidence based, they do involve a degree of subjectivity. However, studies have shown high interrater agreement of these assessment tools [41]. Lastly, UK search engine settings were used for this analysis; it is thus likely that the results of searches in other jurisdictions would be to some extent different. Therefore comparator studies in other jurisdictions would be informative.

This novel study identifies several objective deficiencies in both the quality and readability of online COVID-19 information. Much of the content is difficult to understand for a substantial proportion of the population, and the quality of that information is variable and often poor. A knowledge of these quality and readability assessment tools can assist clinicians in identifying the best sources of online information for patients, as well as providing a means through which authors can optimise the quality and readability of patient information prior to publication. Furthermore, ensuring health information quality and readability protects the rights of the individual to freedom of expression and opinion, as well as safeguarding against false information, particularly in times of public health emergency.

## Data Availability

The data that support the findings of this study are available from the corresponding author [NEWK], upon reasonable request

## Abbreviations

ANOVA: Analysis of variance
COVID-19: Coronavirus disease 2019
FKGL: Flesch-Kincaid Grade Level
FRES: Flesch Reading Ease Score
GFI: Gunning-Fog Index
HON: Health On the Net Foundation
HONcode: Health On the Net Foundation Code of Conduct
JAMA: Journal of the American Medical Association
SARS-CoV-2: Severe acute respiratory syndrome coronavirus 2
